# *ABTB2* Regulatory Variant as Predictor of Epirubicin-Based Neoadjuvant Chemotherapy in Luminal A Breast Cancer

**DOI:** 10.3389/fonc.2020.571517

**Published:** 2020-09-25

**Authors:** Yajie Gong, Nanlin Hu, Li Ma, Wentong Li, Xiang Cheng, Yi Zhang, Ying Zhu, Yang Yang, Xiating Peng, Danyi Zou, Jianbo Tian, Lan Yang, Shufang Mei, Xiaoyang Wang, Chun-han Lo, Jiang Chang, Tieying Hou, Hong Zhang, Binghe Xu, Rong Zhong, Peng Yuan

**Affiliations:** ^1^Department of Epidemiology and Biostatistics and Ministry of Education Key Lab of Environment and Health, School of Public Health, Tongji Medical College, Huazhong University of Science and Technology, Wuhan, China; ^2^Department of Clinical Laboratory Medicine, Guangdong Provincial People's Hospital and Guangdong Academy of Medical Sciences, Guangzhou, China; ^3^Department of Medical Oncology, National Cancer Center, National Clinical Research Center for Cancer, Cancer Hospital, Chinese Academy of Medical Sciences and Peking Union Medical College, Beijing, China; ^4^Department of Breast Center, The Fourth Hospital of Hebei Medical University, Shijiazhuang, China; ^5^Department of Hepatobiliary Surgery, Union Hospital, Tongji Medical College, Huazhong University of Science and Technology, Wuhan, China; ^6^Department of Epidemiology, Harvard T.H. Chan School of Public Health, Boston, MA, United States; ^7^Department of Pathology, Memorial Sloan Kettering Cancer Center, New York, NY, United States; ^8^Department of VIP Medical Services, National Cancer Center, National Clinical Research Center for Cancer, Cancer Hospital, Chinese Academy of Medical Sciences and Peking Union Medical College, Beijing, China

**Keywords:** luminal A breast cancer, *ABTB2*, neoadjuvant chemotherapy, epirubicin resistance, single nucleotide polymorphism

## Abstract

**Background:** Epirubicin combined with docetaxel is the cornerstone of neoadjuvant chemotherapy (NAC) for breast cancer. The efficacy of NAC for luminal A breast cancer patients is very limited, and single nucleotide polymorphism is one of the most important factors that influences the efficacy. Our study is aimed to explore genetic markers for the efficacy of epirubicin combined with docetaxel for NAC in patients with luminal A breast cancer.

**Methods:** A total of 421 patients with two stages of luminal A breast cancer were enrolled in this study from 2 centers. Among them 231 patients were included in the discovery cohort and 190 patients are in the replication cohort. All patients received epirubicin 75 mg/m^2^ and docetaxel 75 mg/m^2^ on day 1, in a 21-day cycle, a cycle for 2–6 cycles. Before treatment, 2 ml of peripheral blood was collected from each patient to isolate genomic DNA. Fourteen functional variants potentially regulating epirubicin/docetaxel response genes were prioritized by CellMiner and bioinformatics approaches. Moreover, biological assays were performed to determine the effect of genetic variations on response to chemotherapy.

**Results:** The patients carrying rs6484711 variant A allele suffered a poor response to epirubicin and docetaxel for NAC (OR = 0.37, 95% CI: 0.18–0.74, *P* = 0.005) in combined stage. Moreover, expression quantitative trait loci (eQTL) analyses and luciferase reporter assays revealed that rs6484711 A allele significantly increased the expression of *ABTB2*. Subsequent biological assays illustrated that upregulation of *ABTB2* significantly reduced the apoptosis rate of breast cancer cells and enhanced the chemo-resistance to epirubicin.

**Conclusions:** Our study demonstrated rs6484711 polymorphism regulating *ABTB2* expression might predict efficacy to epirubicin based NAC in luminal A breast cancer patients. These results provided valuable information about potential role of genetic variations in individualized chemotherapy.

## Background

Breast cancer is the most common malignant cancer and the second leading cause of death among women worldwide (1). Luminal A is the most common subtype of breast cancer, mainly manifested as estrogen receptor (ER) positive, human epidermal growth factor receptor 2 (HER-2) negative, low Ki-67 (2), accounting for 50.6–71% of all breast cancer patients (3, 4). Luminal A type is characterized by sensitivity to endocrine therapy and relatively insensitive to chemotherapy, so for this type of metastatic and postoperative patients, endocrine therapy is the mainstay choice.

The primary purpose of neoadjuvant chemotherapy (NAC) is to reduce the tumor volume so that patients who will suffer mastectomy can gain breast-conserving opportunities, and patients who cannot undergo surgery can obtain surgical opportunities. Recent studies have shown that pathological complete response (pCR) after neoadjuvant is associated with long-term prognosis, especially in HER-2 positive and triple-negative breast cancer (TNBC). Patients with pCR have a lower long-term recurrence rate, but this result does not seem to be in line with patients with luminal A breast cancer (5). Therefore, the main purpose of the neoadjuvant therapy for patients with luminal A breast cancer is still to create surgical opportunities and reduce the range of surgery. Currently, neoadjuvant therapy for luminal A patients includes NAC and neoadjuvant endocrine therapy. NAC is still the preferred treatment (6, 7). The chemotherapy regimens of this type of patients are mainly anthracycline and taxane, but the effective rate is only 13–14.1% (8, 9), sometimes even lower than the effective rate of neoadjuvant endocrine therapy (10), and patients who do not respond to chemotherapy still suffer painful side effects. Therefore, it's a dilemma to choose chemotherapy or endocrine therapy as neoadjuvant therapy for patients who desired to have breast-conserving surgery or get access to surgery.

Among the factors that affect the therapeutic efficacy, the role of individual differences cannot be ignored, and genetic variation plays an important role. Single nucleotide polymorphism (SNP) is a type of common genetic variation. In recent years, studies have found that key genes located in key pathways such as cell proliferation, apoptosis, and DNA repair are related to the efficacy of paclitaxel and epirubicin. Variation in the regulatory and coding regions can significantly affect gene expression or protein function, and may affect the efficacy of taxanes and anthracyclines (11–13).

Based on this, this study uses the change of tumor volume under image monitoring as the main observational endpoint, aiming to explore the individual genetic variation affecting the efficacy of NAC based on anthracycline in luminal A breast cancer and clarify its possible mechanisms.

## Methods

### Patients

This study recruited patients with Luminal A breast cancer who were ≥18 years old, staged T_1−4_N_1−3_M_0_ [American Joint Committee on Cancer (AJCC) 7th edition], diagnosed by core needle aspiration immunohistochemistry (IHC), and were given epirubicin 75 mg/m^2^ and docetaxel 75 mg/m^2^ on day 1, 21 days a cycle for 2–6 cycles, adverse events (AEs) were graded according to Common Terminology Criteria for Adverse Events (CTCAE) version 4.0. Two milliliter of venous blood of the patient was collected before treatment and stored in a minus 80°C medical refrigerator. Luminal A breast cancer is defined as ER positive, progesterone receptor (PR) ≥ 20%, HER-2 negative (HER-2 negative is defined as IHC 0–1, or IHC 2 with Fluorescence *in situ* hybridization(FISH) negative), ki-67 <14% (14). Tumor response was evaluated by magnetic resonance imaging (MRI) before treatment and every 2 cycles according to the Response Evaluation Criteria In Solid Tumors (RECIST) version 1.1. Patients with complete response (CR) and partial response (PR) were divided into the effective group, while patients with stable disease (SD) and progression disease (PD) were divided into the ineffective group.

Patients from January 1, 2015 to December 31, 2016 were enrolled in the discovery cohort (DC) from the Cancer Hospital, Chinese Academy of Medical Sciences, and patients from the Fourth Hospital of Hebei Medical University and the Cancer Hospital of the Chinese Academy of Medical Sciences from January 1, 2017 to December 31, 2017 and were included as the replication cohort (RC).

This project was approved by Ethnics Committee of Cancer Hospital, Chinese Academy of Medical Sciences and the Fourth Hospital of Hebei Medical University. This study was performed in accordance with the Declaration of Helsinki. All patients signed an informed consent form.

### Selection of Candidate SNPs

We firstly extracted epirubicin/docetaxel response genes that their expression were correlated with resistance/sensitivity of epirubicin (NCI No. 256942) or docetaxel (NCI No. 628503) in the CellMiner database (http://discover.nci.nih.gov/cellminer/) (15). According to the criteria that expression of genes with a Pearson's correlation coefficient to growth inhibition values (GI50, a measurement index of cell line sensitivity) below −0.4 or above 0.4, we obtained 284 genes for epirubicin and 228 genes for docetaxel. Considering redundancy, 511 genes were retrieved and considered as potential biomarkers of resistance or sensitivity. Then, we acquired all SNPs located in 5 kb upstream and genes with minor allele frequency (MAF) >0.05 among Han Chinese from the 1000 Genomes Project (http://www.1000genomes.org/). Finally, ANNOVAR software tool (16) was applied to annotate the functions of genetic variations and 14 SNPs were prioritized as candidate regulatory SNPs for the following genotyping. The information of candidate SNPs were shown in Supplementary Table 1.

### Genotyping

Genomic DNA was isolated from 2 ml of peripheral blood lymphocytes using the Relax Gene Blood DNA System DP319-02 (Tiangen, Beijing, China). Fourteen candidate variants were genotyped using the TaqMan Openarray assay system in stage 1 of the study. Candidate SNP was replaced by its highly linkage disequilibrium (LD) SNP for genotyping, when probe design failure or interference with other polymerase chain reaction primers in the reaction system. Each 96-sample array chip contained one NTC (without template DNA) and one duplicated sample to verify the genotyping accuracy. The average call rate for all the candidate SNPs genotyped was >95% and the concordance rate for the duplicate sets was 100%. In validated stage, the promising SNPs were analyzed by a TaqMan real-time polymerase chain reaction (PCR) assay (Applied Biosystems, Foster City, CA) or directly sequencing, without knowledge of the clinical outcomes of the subjects. Approximately 5% of the random samples from effective group and ineffective group were genotyped twice, and the results were in 100% concordance.

### Construction of Plasmids

DNA fragments containing rs6484711[G] or rs6484711[A] were subcloned into pGL3-Basic vector (Promega, USA), respectively. The full-length cDNA of *ABTB2* was subcloned into the pcDNA3.1(+) vector (Invitrogen, USA). All recombinant plasmids were synthesized and verified for sequence by Genewiz Company (Suzhou, China).

### Cell Culture

Human MCF-7 and T-47D breast cancer cell lines were purchased from the China Center for Type Culture Collection (Wuhan, China). Cell lines were cultured in Dulbecco's Modified Eagle's Medium (DMEM) or Roswell Park Memorial Institute (RPMI) 1640 Medium (Gibco, USA) supplemented with 10% fetal bovine serum (FBS; Gibco, USA) and 1% antibiotics (100 U/mL penicillin and 0.1 mg/mL streptomycin) at 37°C in a humidified atmosphere of 5% CO_2_. DNA sequencing using an Applied Biosystems AmpF/STR Identifier kit was performed to test all cell lines routinely and cell lines were tested for free from mycoplasma infection (MycoAlert, USA).

### Dual Luciferase Reporter Gene Assays

The luciferase reporter assay was performed using a dual-Luciferase Reporter Kit (Promega) according to the manufacturer's recommendations. MCF-7 and T-47D cells were seeded in 96-well plates for 24 h. Subsequently, constructed vectors with different alleles of rs6484711 and negative control pGL3-Basic vector were transiently co-transfected with pRL-TK Renilla luciferase vector (Promega) into the cells, using Lipofectamine 3000 Reagent (Invitrogen), respectively. Luciferase activity was measured after transfection for 24 h. For each sample, relative activity was calculated by the ratio of firefly to renilla luciferase signal. Three independent experiments were performed, and triplicate wells were transfected in each experiment.

### Expression Quantitative Trait Loci (eQTL) Analysis

Total RNA was extracted from tumor tissues from 65 luminal breast cancer patients recruited at the Union Hospital, Huazhong University of Science and Technology using TRIzol LS Reagent (Invitrogen, Carlsbad, CA, USA) according to the protocol and was immediately reverse transcribed to cDNA by using the PrimeScript RT Master Mix (TaKaRa, Kyoto, Japan). Quantitative PCR (qPCR) was performed with Power SYBR Green PCR Master Mix (TaKaRa), following the SYBR-green method22 on the ABI 7900 real-time PCR System (Applied Biosystems, Foster City, CA, USA). *ABTB2* expression was normalized to that of GAPDH. The primers used in qPCR were as follows: *ABTB2*-F:5′-TGCGGCAAGAACGCCAATG-3′ and *ABTB2*-R:5′-ACGGGAGACCAAGTCACTCAGCT-3′. Each sample for a given gene was analyzed in duplicate to reduce confounding variance. DNA was also extracted from tumor tissues, and genotyping of rs6484711 was performed as described above. We also downloaded mRNA data and SNPs genotyping information of breast cancer subjects from the Cancer Genome Atlas (TCGA) database and applied MACH-Admix software to imputed rs6484711 genotype using LD and haplotype information from the 1000 Genomes data (phase I version 3) as a reference set.

### Cell Viability Assay

MCF-7 and T-47D cells were seeded and transfected with constructed vector containing full-length *ABTB2* cDNA or pcDNA3.1(+) vector (control) in 12-well flat-bottomed plates (1 × 10^5^ cells per well), respectively. After incubation for 24 h, cells were harvested by trypsin digestion and subsequently seeded in 96-well plates overnight at 37°C, and each well-contained 7 × 10^3^ cells per well. Cells were treated with different concentrations of epirubicin (MedChemexpress) in the medium for 24, 48, and 72 h. At each time point, cell viability was measured using the CCK-8 kit (Dojindo, Tokyo, Japan), according to the manufacturer's instructions.

### Fluorogenic Caspase Activity Assay

For apoptosis assay, cells were treated as described for the CCK-8 assay with 1.0 μM epirubicin for 24 h. Cells in 96-well plate were rinsed with ice-cold phosphate-buffered saline and subsequently cell lysis buffer was added (#7018, Cell Signaling Technology, Danvers, MA, USA). Caspase activity assay (Caspase-3 Activity Assay Kit #5723, Cell Signaling Technology, Danvers, MA, USA) was performed according the manufacturer's instructions. Fluorescence was measured with an excitation wavelength of 380 nm and emission wavelength at 460 nm and expressed in relative fluorescence units (RFU).

### Statistical Analysis

Pearson's χ^2^-test (for categorical variables) and Student's *t*-test (for continuous variables) were used to examine differences between groups with different clinical outcomes in the distribution of demographic characteristics. The distributions of genotype frequencies between groups with different clinical outcomes were calculated by Pearson's χ^2^-test. The association between candidate SNPs and response to NAC were estimated by odds ratios (ORs) and their confidence intervals (95% CIs) using unconditional multivariate logistic regression analysis after adjustment for clinical factors. All *P*-values were two-sided, and differences with *P*-values of < 0.05 were considered statistically significant. All statistical analyses were conducted by Statistic Analysis System software (version 8.2, SAS Institute, Cary, NC).

## Results

### Demographic Characteristics of Participants in Two Stage Cohort Studies

From January 1, 2015 to December 31, 2016, 231 patients were included in DC from the Cancer Hospital of the Chinese Academy of Medical Sciences, and 190 patients were recruited in the RC from January 1, 2017 to December 31, 2017, including 90 patients from the Fourth Hospital of Hebei Medical University and 100 patients from the Cancer Hospital of the Chinese Academy of Medical Sciences. For a total of 421 patients, 65.8% (152/231) and 71.1% (135/190) had axillary lymph node metastasis and the effective rate was 77.5% (179/231) and 73.2% (139/190) in the DC and RC group, respectively. 75.5% (318/421) patients in total, 77.5% (179/231) in DC and 73.2% (139/190) in RC had effective response, including CR and PR. 24.5% (103/421) patients in total, 22.5% (52/231) in DC and 26.8% (51/190) in RC got no response which means SD and PD. The demographic characteristics of the patients in the two-stage cohort study were presented in Table 1. Briefly, in stage 1, the effective rate of neoadjuvant treatment was 77.5%. The median age of the series was 47.9 years in effective group and 50.3 years in ineffective group, and the distribution of age were well-matched between two groups (*P* = 0.112). One hundred and fifty patients presented infiltrating ductal carcinoma (IDC)-I, 36 with IDC-II and 43 with IDC-III, and the remaining 2 cases corresponded to pathologic type unknown. No statistically significant difference was found in menopausal status (*P* = 0.337), clinical T-stage (*P* = 0.702), lymph node metastasis (*P* = 0.368), myelosuppression (*P* = 0.085) and gastrointestinal side effects (*P* = 0.546) between effective group and ineffective group. Moreover, the effective rate of 72.4% was observed after neoadjuvant treatment in stage 2. Except for myelosuppression, similar distributions of these characteristics between the two groups were also observed (*P* > 0.05).

**Table 1:** Characteristics of participants in the two-stage study.

**Variables**	**Stage 1 (No**. **=** **231)**				**Stage 2 (No**. **=** **190)**			
	**Effective group^**a**^**	**Ineffective group^**b**^**	*****χ***^2^/*t***	***P***	**Effective group^**a**^**	**Ineffective group^**b**^**	*****χ*****^2^**/*t***	***P***
	**No. (%)**	**No. (%)**			**No. (%)**	**No. (%)**		
Total	179 (77.5)	52 (22.5)			139 (73.2)	51 (26.8)		
Age (mean ± SD)	47.9 ± 9.60	50.3 ± 8.67	1.60	0.112	47.1 ± 9.94	49.3 ± 10.27	1.37	0.174
Menopausal status			0.92	0.337			3.31	0.069
Postmenopausal	66 (36.9)	23 (44.2)			48 (34.5)	25 (49.0)		
Premenopausal	113 (63.1)	29 (55.8)			91 (65.5)	26 (51.0)		
Clinical T-stage			1.42	0.702			3.76	0.289
T1	25 (14.0)	8 (15.4)			20 (14.4)	9 (17.6)		
T2	115 (64.2)	29 (55.8)			70 (50.4)	31 (60.8)		
T3	32 (17.9)	12 (23.1)			34 (24.5)	9 (17.6)		
T4	7 (3.9)	3 (5.8)			15 (10.8)	2 (3.9)		
Lymph-node status			0.81	0.368			2.93	0.087
cN+	116 (68.2)	36 (75.0)			91 (65.5)	40 (78.4)		
cN0	54 (31.8)	12 (25.0)			48 (34.5)	11 (21.6)		
Myelosuppression			2.96	0.085			4.15	0.042
Yes	126 (70.4)	30 (57.7)			83 (59.7)	22 (43.1)		
No	53 (29.6)	22 (42.3)			56 (40.3)	29 (56.9)		
Gastrointestinal side effects			0.37	0.546			0.27	0.602
Yes	98 (54.7)	26 (50.0)			111 (81.0)	43 (84.3)		
No	81 (45.3)	26 (50.0)			26 (19.0)	8 (15.7)		
Histological diagnosis			7.86	0.049			1.71	0.790
IDC-I	111 (62.0)	39 (75.0)			9 (6.5)	2 (3.9)		
IDC-II	34 (19.0)	2 (3.8)			40 (28.8)	18 (35.3)		
IDC-III	33 (18.4)	10 (19.2)			51 (36.7)	20 (39.2)		
ILC	–	–			9 (6.5)	2 (3.9)		
Other	1 (0.6)	1 (1.9)			30 (21.6)	9 (17.6)		

pCR, pathologic complete response; cCR, clinical complete response; PR, partial response; SD, stable disease; PD, progressive disease; IDC, Infiltrating ductal carcinoma; ILC, Infiltrating lobular carcinoma; Others (micropapillary and tubular carcinomas and pathologic type unknown).

a Effective group contained participants with response of complete response or partial response to neoadjuvant chemotherapy;

b *Ineffective group contained participants with stable disease and progression disease to neoadjuvant chemotherapy*.

### Associations of Candidate SNPs With Response to NAC

In stage 1, as shown in Table 2, the associations between SNPs with therapeutic effect were evaluated by unconditional logistic regression after adjusting for age, menopause status, lymph node metastasis and side effects. The CC genotype of rs1925368, which was in complete LD with rs6484711, showed significant association with poor response to chemotherapy (recessive model: OR = 0.38, 95% CI = 0.15–0.96, *P* = 0.041), compared to the CG and GG genotypes. The TT genotype of rs10747780 (complete LD with rs184301136) was also associated with decreased effect (OR = 0.43, 95% CI = 0.21–0.87, *P* = 0.019), compared with the CC genotype. Therefore, the two SNPs were selected for the following validation study.

**Table 2:** Association of candidate variants with therapeutic effect of neoadjuvant chemotherapy in the discovery stage.

**SNPs**	**Call rate (%)**	**HT vs. HW**		**HV vs. HW**		**Additive model**		**Recessive model**		**Dominant model**	
		**OR (95% CI)^***a***^**	***P***	**OR (95% CI)^***a***^**	***P***	**OR (95% CI)^***a***^**	***P***	**OR (95% CI)^***a***^**	***P***	**OR (95% CI)^***a***^**	***P***
rs11591030	99.13	1.43 (0.58–3.52)	0.438	–	–	1.54 (0.65–3.65)	0.332	–	–	1.50 (0.61–3.68)	0.376
rs1551655	97.40	0.90 (0.37–2.19)	0.822	0.21 (0.04–1.17)	0.075	0.65 (0.34–1.24)	0.189	0.21 (0.04–1.219)	0.077	0.72 (0.32–1.60)	0.416
rs16970163	98.70	1.06 (0.52–2.18)	0.875	1.13 (0.12–11.09)	0.918	1.06 (0.56–2.01)	0.857	1.11 (0.11–10.71)	0.931	1.06 (0.53–2.15)	0.863
rs201004	97.84	1.19 (0.57–2.48)	0.650	–	–	1.34 (0.67–2.68)	0.412	–	–	1.27 (0.61–2.64)	0.531
rs232835	99.13	1.09 (0.52–2.29)	0.823	–	–	1.09 (0.52–2.29)	0.823	–	–	1.09 (0.52–2.29)	0.823
rs3810919	98.27	1.47 (0.73–2.96)	0.278	2.56 (0.30–21.90)	0.391	1.51 (0.82–2.78)	0.189	2.19 (0.26–18.44)	0.469	1.54 (0.78–3.04)	0.218
rs730870	98.70	0.83 (0.39–1.79)	0.635	0.99 (0.34–2.84)	0.980	0.96 (0.58–1.59)	0.885	1.11 (0.44–2.81)	0.820	0.86 (0.41–1.81)	0.696
rs828095	98.70	1.23 (0.61–2.48)	0.573	–	–	1.656 (0.83–2.92)	0.166	–	–	1.42 (0.71–2.85)	0.325
rs7366009	100.00	1.34 (0.65–2.78)	0.428	0.58 (0.13–2.61)	0.480	1.05 (0.59–1.88)	0.875	0.53 (0.12–2.32)	0.398	1.21 (0.61–2.40)	0.593
rs402645	100.00	0.75 (0.35–1.58)	0.449	0.59 (0.23–1.52)	0.271	0.76 (0.48–1.22)	0.258	0.70 (0.30–1.60)	0.395	0.70 (0.35–1.42)	0.326
rs11145930	100.00	1.09 (0.45–2.61)	0.852	–	–	1.20 (0.52–2.76)	0.664	–	–	1.15 (0.48–2.74)	0.756
rs1925368^**b**^	99.13	0.88 (0.41–1.90)	0.779	**0.35 (0.13–1.00)**	**0.050**	0.65 (0.39–1.09)	0.100	**0.38 (0.15–0.96)**	**0.041**	0.74 (0.36–1.51)	0.405
rs10747780^**b**^	98.27	**0.43 (0.21–0.87)**	**0.019**	1.05 (0.21–5.24)	0.955	0.67 (0.39–1.13)	0.133	1.64 (0.35–7.75)	0.530	**0.47 (0.24–0.94)**	**0.033**
rs1144943	98.27	1.20 (0.59–2.44)	0.612	0.57 (0.19–1.73)	0.322	0.89 (0.53–1.49)	0.663	0.53 (0.18–1.51)	0.232	1.04 (0.54–2.03)	0.902

HW, wild type homozygote; HT, heterozygote; HV, variant homozygote; OR, odds ratio; 95% CI, 95% confidence interval. The significant results were in bold.

a *Data were calculated by unconditional logistic regression model after adjusting for age, menopause status, lymph node metastasis, myelosuppression, gastrointestinal side effects. ^b^rs1925368 stands for rs6484711 (r^2^ = 1) and rs10747780 stands for rs184301136 (r^2^ = 0.97) from the 1000 Genomes Project*.

In the validation stage, only the rs6484711 variant A allele revealed an association with poor therapeutic effect and had an OR of 0.35 (95% CI = 0.13–0.91, *P* = 0.032), which coincided with the result in stage 1 (Table 3). The combined analysis in Table 4 also exhibited allele A of rs6484711 conferred significantly poor response to chemotherapy, compared with the GG genotype (OR = 0.37, 95% CI = 0.18–0.74, *P* = 0.005).

**Table 3:** Association analyses between variants and therapeutic effect of neoadjuvant chemotherapy in the replication stage.

**SNPs**	**HT vs. HW**		**HV vs. HW**		**Additive model**		**Dominant model**		**Recessive model**	
	**OR (95% CI)^***a***^**	***P***	**OR (95% CI)^***a***^**	***P***	**OR (95% CI)^***a***^**	***P***	**OR (95% CI)^****a****^**	***P***	**OR (95% CI)^***a***^**	***P***
rs6484711	0.63 (0.28–1.39)	0.253	**0.35 (0.13–0.91)**	**0.032**	**0.59 (0.37–0.96)**	**0.033**	0.53 (0.25–1.11)	0.094	0.46 (0.20–1.05)	0.065
rs184301136	1.81 (0.90–3.65)	0.095	–	–	1.81 (0.90–3.65)	0.095	1.82 (0.91–3.66)	0.092	–	–

HW, wild type homozygote; HT, heterozygote; HV, variant homozygote; OR, odds ratio; 95% CI, 95% confidence interval. The significant results were in bold.

a *Data were calculated by unconditional logistic regression model after adjusting for age, menopause status, lymph node metastasis, myelosuppression, gastrointestinal side effects*.

**Table 4:** Association analyses between rs6484711 and therapeutic effect of neoadjuvant chemotherapy in the combined study.

**SNP**	**Genotypes**	**Effective/ineffective^***a***^**	**OR (95% CI)^***b***^**	***P***
rs6484711	GG	122/30	1.00	
	GA	155/49	0.80(0.47–1.37)	0.416
	AA	36/23	0.37(0.18–0.74)	0.005
	Additive		0.63(0.44–0.89)	0.010
	Dominant		0.66(0.40–1.10)	0.109
	Recessive		0.42(0.23–0.78)	0.006

OR, odds ratio; 95% CI, 95% confidence interval.

a Effective group contained participants with response of CR or PR to neoadjuvant chemotherapy; Ineffective group contained participants with SD and PD to neoadjuvant chemotherapy.

b *Data were calculated by unconditional logistic regression model after adjusting for age, menopause status, lymph node metastasis, myelosuppression, gastrointestinal side effects*.

### The rs6484711 Influences the Promoter Activity of *ABTB2*

**Figure 1:**
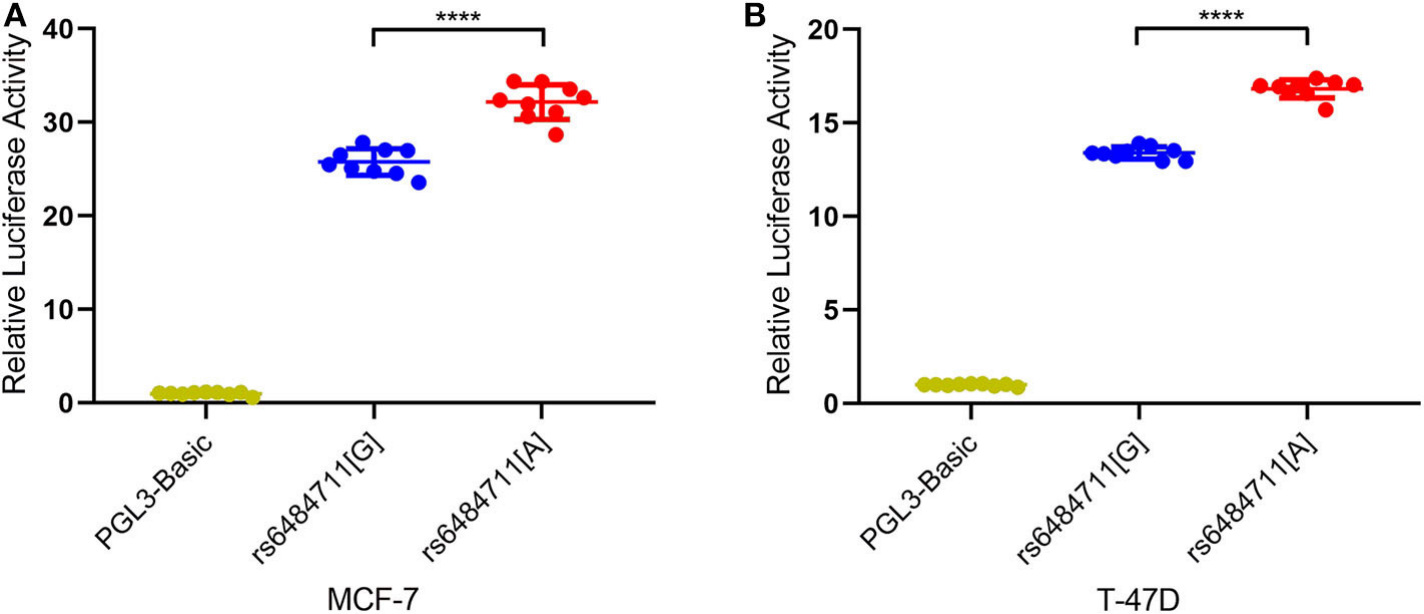
Reporter gene expression driven by different rs6484711 alleles in MCF-7 **(A)** and T-47D **(B)** cells. Luciferase activities were shown as fold changes relative to luciferase expression in cells transfected with empty vectors (pGL3-Basic). All constructs were cotransfected with PRL-TK to standardize transfection efficiency. Data shown were the mean ± S.D. from three independent experiments, each had three replicates. ****P < 0.0001.

Since variant in the non-coding region might be implicated in gene expression regulation, we then performed dual-luciferase reporter assays with pGL3-Basic firefly luciferase expression vector containing allele-different fragments harboring rs6484711. Transfection of these plasmids into MCF-7 and T-47D cells resulted in significantly different relative luciferase activity (Figures 1A,B), with the rs6484711[A] allele having higher luciferase activity compared with the rs6484711[G] allele (*P* < 0.0001 in both MCF-7 and T-47D cells). These results indicated that rs6484711 may act promoter activity regulating transcription of *ABTB2*.

### Identification of rs6484711 Influencing Expression of *ABTB2*

**Figure 2:**
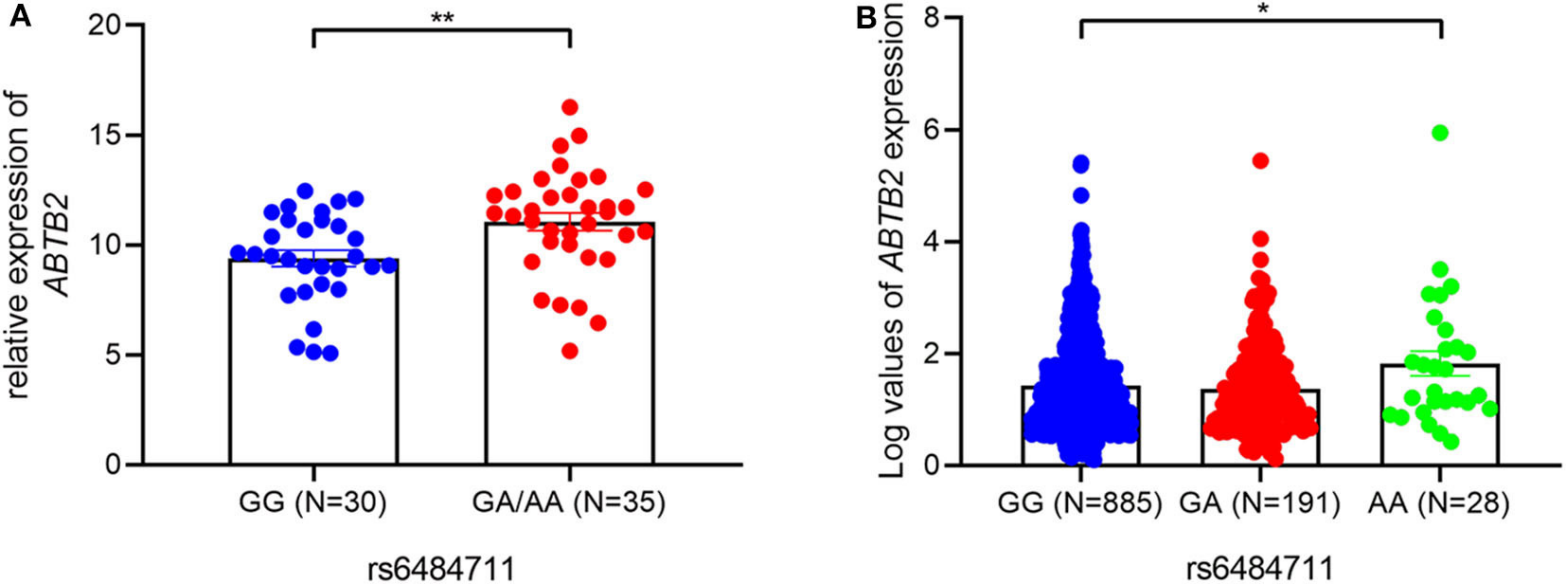
The eQTL analyses of rs6484711. The associations between rs6484711 genotypes and *ABTB2* levels in 65 BC tissues **(A)** and 1104 samples in TCGA **(B)**. *ABTB2* mRNA levels were relative to GAPDH using qRT-PCR in our samples and were represented by a log transformation of fragments per kilobase of exon per million fragments mapped (FPKM) value in TCGA data. The rs6484711[GA] and [AA] genotypes had significantly higher *ABTB2* mRNA than the rs6484711[GG] genotype (*P = 0.030, **P = 0.004). Results were shown as the means ± S.D., and *P*-value was from two-sided *t*-tests.

Furthermore, an eQTL analysis was performed to determine whether rs6484711 correlated with the mRNA expression levels of *ABTB2* gene in luminal A breast cancer tumor. The result showed that rs6484711 significantly affected expression levels of *ABTB2* (*P* = 0.004, Figure 2A). Consistent with the result, we also observed notably differential expression of *ABTB2* among individuals carrying different genotypes from the TCGA (Figure 2B, *P* = 0.030). Thus, patients carrying the GA and AA genotypes of rs6484711 have a significantly higher *ABTB2* expression than those with GG genotype.

### Effect of *ABTB2* on the Epirubicin Resistance in Breast Cancer Cells

**Figure 3:**
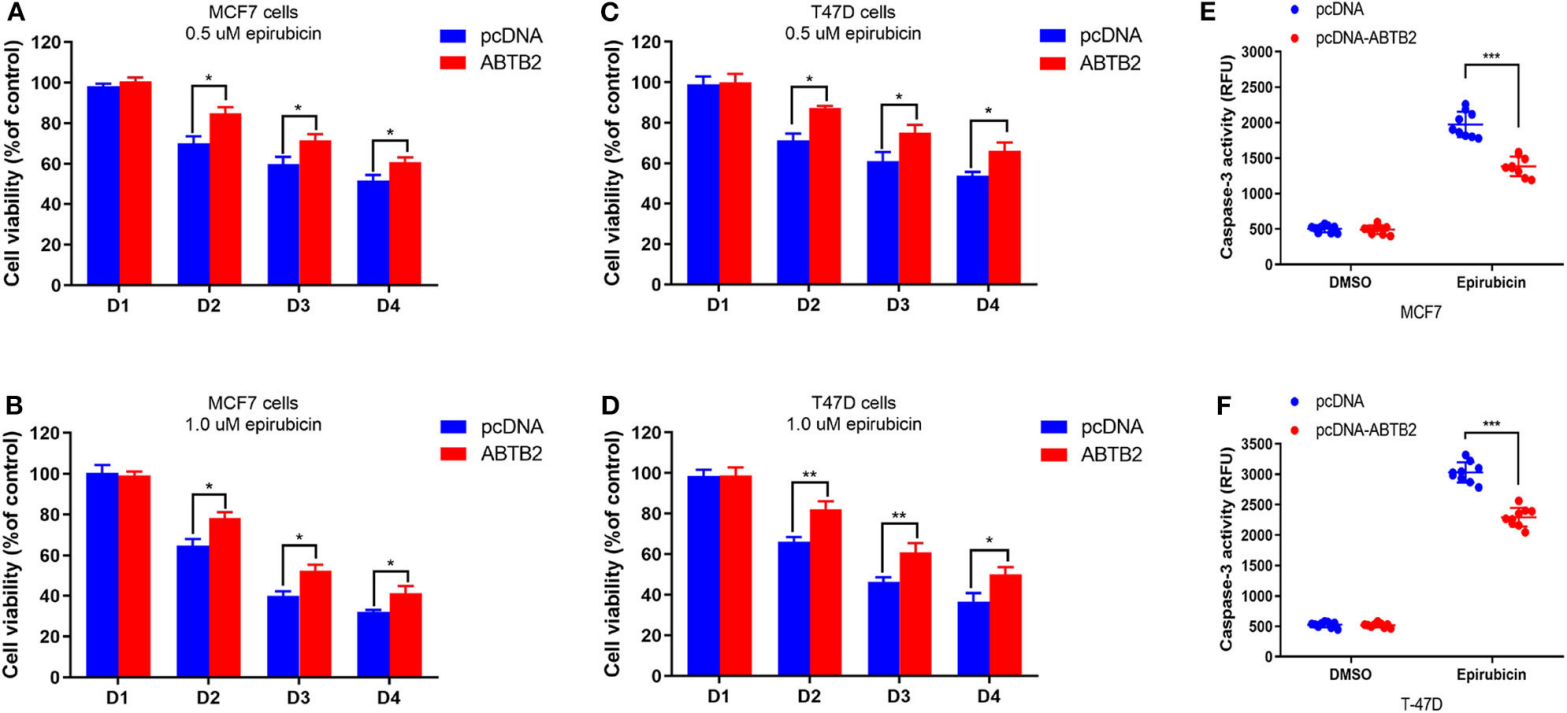
Effects of *ABTB2* on cell viability in epirubicin-treated MCF-7 and T-47D cells. Twenty-four hours after transfection with pcDNA-*ABTB2* or control vectors, MCF-7 **(A,B)** and T-47D **(C,D)** cells were treated with epirubicin (0.5 and 1.0 μM, respectively) for 0, 24, 48, and 72 h. **(E,F)** Test of caspase-3 activity in *ABTB2*-overexpression cells or control cells treated with 1.0 μM epirubicin in both the two breast tumors cell lines. Experiments were repeated three times with mean ± S.D. *P < 0.05, **P < 0.01, ***P < 0.001.

To investigate the effect of *ABTB2* on chemo-sensitivity *in vitro*. MCF-7 and T-47D cells were treated with epirubicin for a certain time after overexpression of *ABTB2*. Upon exposure to 0.5 μM epirubicin, decreased cell viability was showed in MCF-7 cells. However, the rate of cell viability had a significant improvement in *ABTB2*-overexpressed MCF-7 cells, compared with control group (Figure 3A). Moreover, enhanced epirubicin-resistance was more obvious in MCF-7 cells with overexpression of *ABTB2*, when treated with 1.0 μM epirubicin (Figure 3B). Consistent results were also observed in *ABTB2*-overexpressed T-47D cell lines from D2 to D4 (Figures 3C,D).

**Figure 4:**
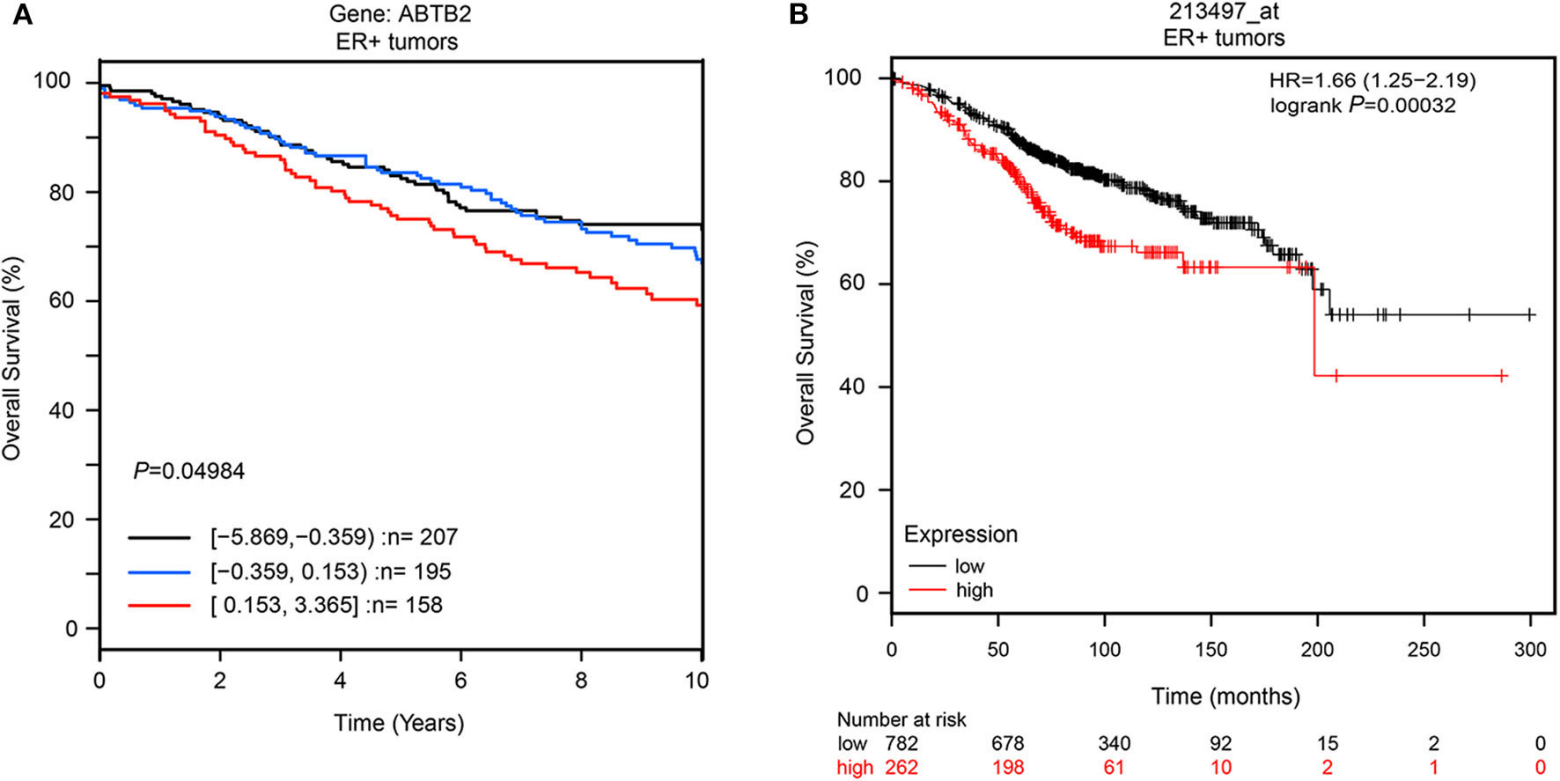
The overall survival in different expression of *ABTB2* in ER-positive breast cancer. We obtain data from two different published database. *ABTB2* expression correlated with overall survival in 560 ER-positive breast cancer patients through GOBO Gene Set Analysis, *P* = 0.049 **(A)**; Patients with high expression of *ABTB2* has significantly shorter overall survival than those with low expression **(B)** *P* = 0.0003.

Furthermore, to explore whether *ABTB2* contributed to the enhanced resistance to epirubicin-induced cell apoptosis, we evaluated the caspase-3 activity in MCF-7 and T-47D cells in the presence of epirubicin. After overexpression of *ABTB2*, the cells were treated with 1.0 μM epirubicin for 24 h, and we detected that epirubicin markedly activated caspase-3 in the two cell lines. But cells transfected with *ABTB2* significantly inhibited the activation of caspase-3, in comparison to control group (Figures 3E,F). Thus, *ABTB2* overexpression obviously enhanced the epirubicin-resistant phenotype of MCF-7 and T-47D cells. In addition, we observed that *ABTB2* expression correlated with overall survival in 560 ER-positive breast cancer patients through GOBO Gene Set Analysis (17) (Figure 4A), and similar result was discovered in another database (18) (Figure 4B), which suggested that *ABTB2* was associated with progression and poor outcomes of ER-positive breast cancer. Therefore, our results indicate that *ABTB2* acting as a drug-resistant protein negatively affects epirubicin-induced cell apoptosis and mediates epirubicin-resistance in the MCF-7 and T-47D cells.

## Discussion

Our study is the first to find that *ABTB2* expression was associated with efficacy in breast patients with luminal A subtype who undergo epirubicin and docetaxel for NAC. In addition, SNP rs6484711 variant A allele tend to increase the expression of *ATBT2* and patients with GA or AA genotype suffer 63% lower effective rate than GG genotype (OR = 0.37, *P* = 0.005). It is suggested that SNP rs6484711 variant A allele could be a potential biomarker in luminal A subtype of breast cancer patients who receive epirubicin and docetaxel for NAC.

Up to now, anthracycline resistance is associated with multiple mechanisms, including alteration in DNA repair, changes in topoisomerase II activity, stemness of tumor cells, and metabolic adaptation (19). Some studies have shown that long non-coding RNA NONHSAT101069, SIRT6 protein, transforming growth factor (TGF-β), fibroblast growth factor receptor (FGFR) 4 rs1966265 and FGFR2 rs2981578 get involved in anthracycline resistance in breast cancer (20–23), as well as other factors. Genetic variations play an important role in regulating drug resistance.

**Figure 5:**
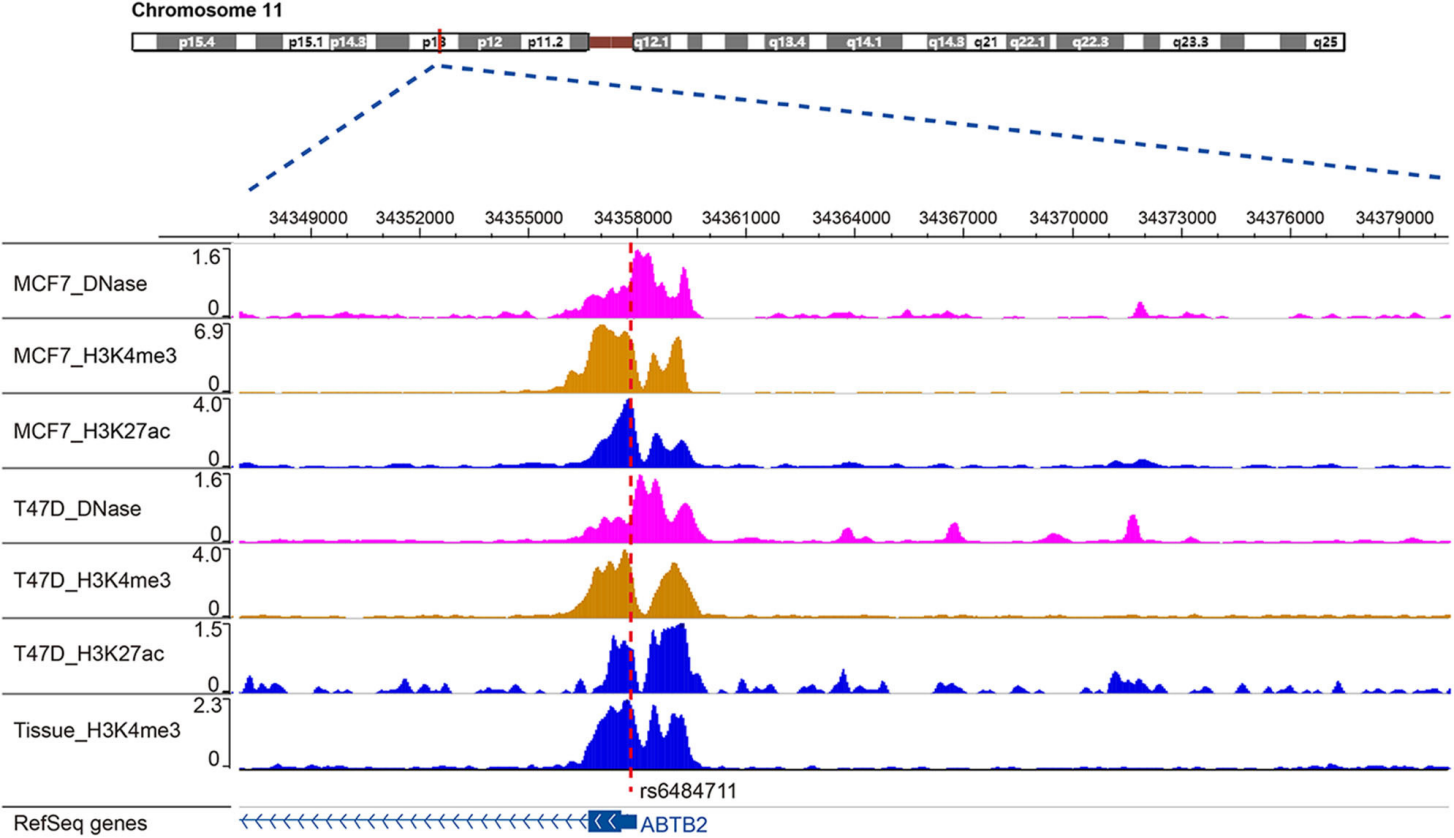
Location of rs6484711 in the ChIP-seq peaks. Through bioinformatics analysis, rs6484711 resides in the ChIP-seq peaks of histone markers (such as H3K4me3 and H3K27ac), as well as active chromatin accessibility.

SNP rs6484711 is located in the 5′UTR of *ABTB2*. Studies have shown that the he majority variants in non-coding regions of genome are often enriched in regulatory elements, which in some cases interfere with gene expression and function (24, 25). Through bioinformatics analysis, rs6484711 resides in the ChIP-seq peaks of histone markers (such as H3K4me3 and H3K27ac), as well as active chromatin accessibility (Figure 5), indicating that rs6484711 may have a potential promoter-like effect. Further eQTL analysis showed that the rs6484711 polymorphism regulated the expression of *ABTB2* (Figure 2A), and the expression of *ABTB2* in the cell line carrying rs6484711 variant A allele increased significantly, which causes resistance to epirubicin. It is suggested that this variant affects the sensitivity to epirubicin in ER positive breast cancer by regulating the expression of *ABTB2*, which is consistent with the conclusion from analysis of TCGA database (Figure 2B). So our research demonstrated that *ABTB2* gene polymorphism can be used as a predictor of efficacy for epirubicin.

The protein encoded by the *ABTB2* gene is ankyrin repeat and BTB/POZ domain-containing protein (26), which participates in the pathological process of Parkinson's disease by affecting the accumulation of α-synuclein (27, 28). While reducing or lacking the expression of *ABTB2* can reduce liver fibrosis (29). It also has the function of regulating cell growth and the degradation of defective proteins, thereby affecting apoptosis (24, 30, 31). The relationship between *ABTB2* and breast cancer has not been reported yet. Our analysis from database revealed that the survival of patients with high expression of *ABTB2* was significantly shortened in ER-positive breast cancer, suggesting that *ABTB2* is closely related to the poor prognosis of ER-positive breast cancer (Figure 4). The mechanism that affects the prognosis is not yet clear. The results of gene ontology (GO) analysis indicate that *ABTB2* may be involved in the cellular response to toxic substances, and for most cytotoxic drugs with different mechanisms they kill tumor cells by inducing apoptosis (32, 33), implying that *ABTB2* is involved in apoptosis. It was found that the activity of cells overexpressing *ABTB2* was significantly increased after given epirubicin (Figures 3A–D), and the activity of caspase-3 in this group of cell lines was significantly inhibited (Figures 3E,F), disclosing that *ABTB2* reduces the sensitivity of ER-positive breast cancer cell lines to epirubicin by inhibiting tumor cell apoptosis. It was the first time that our study reported the relationship between *ABTB2* and breast cancer prognosis, and the mechanism of *ABTB2*-inducing resistance to epirubicin's cytotoxicity.

For now, neoadjuvant endocrine plus targeting therapy is used in more and more patients, and predictive biomarkers of this regimen need to be explored.

## Conclusion

In conclusion, this study was the first to discover that patients with luminal A breast cancer carrying SNP rs6484711 variant A allele at *ABTB2* 5′UTR can significantly reduce the effectiveness of epirubicin combined with docetaxel by regulating the expression of *ABTB2* protein. We also reported that *ABTB2* is related to the resistance of breast cancer cells to epirubicin and the prognosis of ER-positive breast cancer, suggesting that SNP rs6484711 variant A allele can be used as a predictive marker for the efficacy of epirubicin combined with docetaxel for NAC in luminal A breast cancer and *ABTB2* can be used as a prognostic marker for ER-positive breast cancer.

## Data Availability Statement

The raw data supporting the conclusions of this article will be available from the corresponding author by request.

## Ethics Statement

The studies involving human participants were reviewed and approved by Ethics Committee of Cancer Hospital, Chinese Academy of Medical Sciences and the Fourth Hospital of Hebei Medical University. The patients/participants provided their written informed consent to participate in this study.

## Author Contributions

YG: study design, data analysis and interpretation, carry out most of the experiments, and drafting of the manuscript. NH: study design, blood sample acquisition, and drafting of the manuscript. LM, WL, XC, and YZha: study design and blood sample acquisition. YZhu, YY, and XP: blood sample acquisition. DZ, JT, LY, SM, and XW: data acquisition and DNA preparation and genotyping. CL, JC, TH, HZ, and BX: study design and revision of the manuscript. RZ and PY: study concept and design, access to all the data in the study, critical revision of the manuscript for important intellectual content, study supervision, and obtained funding. All authors: read and approved the final manuscript.

## Conflict of Interest

The authors declare that the research was conducted in the absence of any commercial or financial relationships that could be construed as a potential conflict of interest.

## Abbreviations

ER: estrogen receptor
HER-2: human epidermal growth factor receptor 2
NAC: neoadjuvant chemotherapy
pCR: pathological complete response
TNBC: triple-negative breast cancer
SNP: single nucleotide polymorphism
IHC: immunohistochemistry
AJCC: American Joint Committee on Cancer
PR: progesterone receptor
AE: adverse event
CTCAE: Common Terminology Criteria for Adverse Events
FISH: fluorescence *in situ* hybridization
MRI: magnetic resonance imaging
RECIST: Response Evaluation Criteria In Solid Tumors
CR: complete response
PR: partial response
SD: stable disease
PD: progression disease
DC: discovery cohort
RC: replication cohort
GI: growth inhibition
MAF: minor allele frequency
LD: linkage disequilibrium
PCR: polymerase chain reaction
DMEM: Dulbecco's Modified Eagle's Medium
RPMI: Roswell Park Memorial Institute
TCGA: the Cancer Genome Atlas
RFU: relative fluorescence units
ORs: odds ratios
CI: confidential interval
IDC: infiltrating ductal carcinoma
GO: gene ontology.
