# Supplementary material for: ABTB2 Regulatory Variant as Predictor of Epirubicin-Based Neoadjuvant Chemotherapy in Luminal A Breast Cancer
Source: Front Oncol. 2020 Sep 25;10:571517. doi: 10.3389/fonc.2020.571517 (PMC7545368; doi:10.3389/fonc.2020.571517)
Supplement: Supplementary file 1 [file Table_1.DOCX]

Supplementary Table 1. Summary of fourteen candidate SNPs obtained from bioinformatics analyses

| SNP | Chr | Position | Gene | Correlation*^a^* | Location | MAF in CHB | Major/Minor Allele | Predictions | TF(s)*^b^* |
| --- | --- | --- | --- | --- | --- | --- | --- | --- | --- |
| rs11591030 | 1 | 201430285 | *PHLDA3* | -0.497 | intergenic | 0.15 | T/C | Enhancer | 2 TFs |
| rs232835 | 1 | 59042882 | *TACSTD2* | **-0.453** | 5' UTR | 0.15 | T/G | Promoter | 2 TFs |
| rs2154114 | 1 | 223900130 | *CAPN2* | **-0.442** | 5' UTR | 0.20 | A/C | Promoter | 6 TFs |
| rs9791024 | 5 | 64858955 | *PPWD1* | 0.403 | 5' UTR | 0.22 | A/C | Promoter | 11 TFs |
| rs730870 | 5 | 125874993 | *GRAMD3, ALDH7A1* | **-0.483** | intergenic | 0.46 | A/G | Enhancer | 5 TFs |
| rs201004 | 6 | 27804934 | *HIST1H2BN* | 0.489 | downstream | 0.21 | T/C | Enhancer | 4 TFs |
| rs1551655 | 8 | 48873261 | *MCM4* | 0.466 | 5' UTR | 0.12 | A/C | Promoter | 12 TFs |
| rs828095 | 9 | 127961771 | *PPP6C, RABEPK* | 0.417 | intergenic | 0.23 | T/C | Enhancer | 1 TF |
| rs3810919 | 9 | 116037894 | *CDC26, PRPF4* | 0.447 | upstream | 0.32 | C/T | Promoter | 14 TFs |
| rs4400498 | 9 | 139305007 | *SDCCAG3* | 0.407 | 5' UTR | 0.16 | C/T | Promoter | 10 TFs |
| rs6484711 | 11 | 34379503 | *ABTB2* | **-0.418** | 5' UTR | 0.38 | G/A | Promoter | 4 TFs |
| rs184301136 | 12 | 57984923 | *PIP4K2C* | -0.428 | upstream | 0.21 | C/G | Enhancer | 8 TFs |
| rs937282 | 12 | 69201797 | *MDM2* | -0.402 | upstream | 0.27 | C/G | Promoter | 6 TFs |
| rs16970163 | 19 | 35739702 | *LSR* | **-0.418** | 5' UTR | 0.18 | G/A | Promoter | 11TFs |

Abbreviations: chr, chromosome; MAF, minor allele frequency; CHB, Han Chinese in Beijing, China; TF, transcription factor.

*^a^*Pearson's correlation coefficients based on a population size of 60. Significant (with *P*<0.05) positive and negative correlations comparisons in Epirubicin (bold) or Docetaxel treatment are presented in plus or minus signs. *^b^*Number of TFs annotated from ENCODE project.
